# Imaging findings of primary epithelioid sarcoma of adrenal gland: a case report and literature review

**DOI:** 10.3389/fonc.2023.1015258

**Published:** 2023-05-15

**Authors:** Hongyu Yin, Yi Zhang, Linyun Wu, Ji Zhang

**Affiliations:** ^1^ Graduate School of Dalian Medical University, Dalian, Liaoning, China; ^2^ Department of Radiology, Jiangsu Taizhou People’s Hospital, Taizhou, Jiangsu, China; ^3^ Taizhou Polytechnic College, Taizhou, Jiangsu, China

**Keywords:** imaging findings, primary epithelioid sarcoma, adrenal gland, MRI, CT

## Abstract

Epithelioid sarcoma (ES) is a rare soft tissue malignant tumor with an uncertain histogenetic origin. It usually arises in soft tissues of the extremities, while ES in adrenal gland is extremely rare. There is no special clinical manifestation in the early stage, so it may be misdiagnosed and delay the treatment. We reported a 69-year-old male with an adrenal ES. The tumor was completely resected, and two months later, positron emission tomography-computed tomography(PET/CT) noted recurrence at the tumor bed and multiple metastases. The patient has been treated with chemotherapy with good effects. We summarize the radiological findings and immunohistochemical indexes of primary epithelioid sarcoma of adrenal gland, which may be useful to promote disease awareness and help to distinguish among other lesions.

## Introduction

Epithelioid sarcoma (ES), a malignant mesenchymal tumor that occurs in extremities of adolescents, was originally described by Enzinger in 1970 (1). In 1997, Guillion et al. (2) identified proximal ES after observing ES in perineum, genital tract, and pelvis in young people. Recurrence and metastasis are prevalent. Clinically, ES of the adrenal is extremely rare, with a few cases having been documented. In this report, we describe an adrenal ES case with a focus on imaging findings and immunohistochemical indexes to promote disease awareness.

## Case presentation

**Figure 1:**
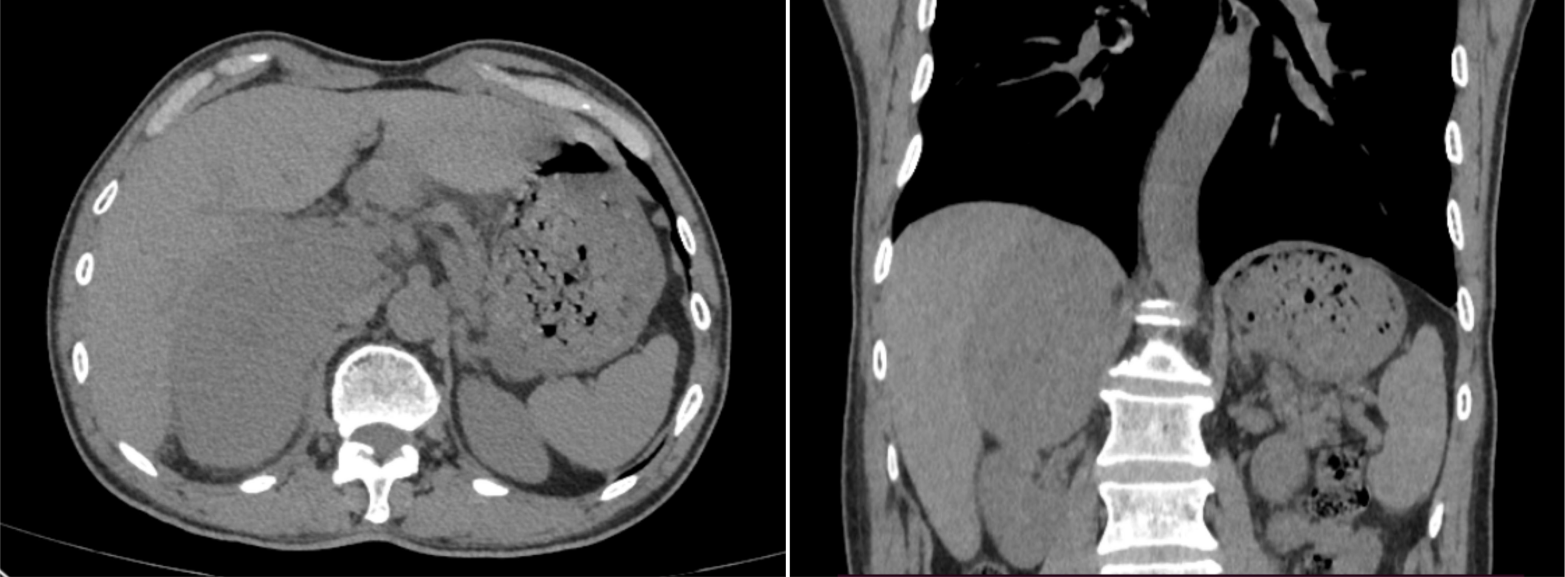
Selected window images of axial, coronal plain abdomen CT scan soft tissue demonstrating a heterogeneous adrenal mass measuring 10.6×6.4×11.0cm, which abutted and compressed the liver parenchyma, right renal and IVC.

**Figure 2:**
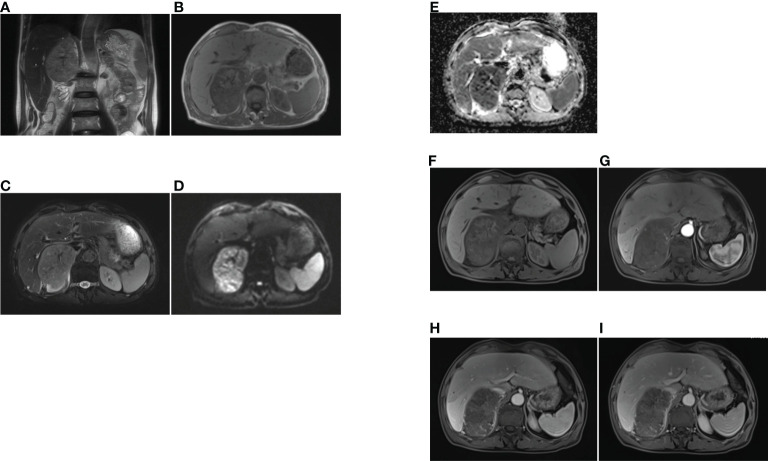
The MRI findings of primary adrenal sarcoma. **(A)** coronal T2-weighted MR image, **(B)** axial T1-weighted MR image, **(C)** axial fat suppression T2-weighted MR image, **(D, E)** The tumor showed heterogeneous high signal intensity on DWI and low signal intensity on ADC map, **(F–I)** axial fat suppression T1-weighted MR imaging **(F)**, and Gadolinium-enhanced T1-weighted MR mages at arterial **(G)**, portal **(H)** and delayed phases **(I)**, the tumor exhibited heterogeneous enhancement and no enhancement in the central scar.

A 69-year-old man with an incidental right adrenal gland mass was admitted to our institution. He had no relevant past medical history, and no family history was identified. Hematological examination did not reveal any abnormal findings. Adrenal function laboratory assay indices were normal, including cortisol, catecholamine and aldosterone. Initial CT scan revealed a round-like, well-defined slightly hypodense lesion in the right adrenal gland region measuring approximately 10.6×6.4×11.0 cm in size, with a CT value of about 34 HU. The right posterior liver, portal vein and upper pole of the right kidney were displayed (**Figure 1**). Right adrenal gland imaging was not conclusive. A follow-up MRI (**Figure 2**) revealed a 10.2×6.5×10.5 cm oval tumor in the right suprarenal region, with a high-low mixed sign on T_1_WI and slightly high signals on fat suppression T_2_WI sequence. A central scar was noted in the lesion, which was hypointense on T_1_WI and T_2_WI. Upon enhancement scan, the lesion exhibited a slight to moderate heterogeneous enhancement with no enhancement in the central scar. Visualization of the right adrenal gland was not clear. There were no significant enlarged lymphnodes or metastatic foci. Gene detection and FDG PET/CT were not carried out due to economic reasons.

**Figure 3:**
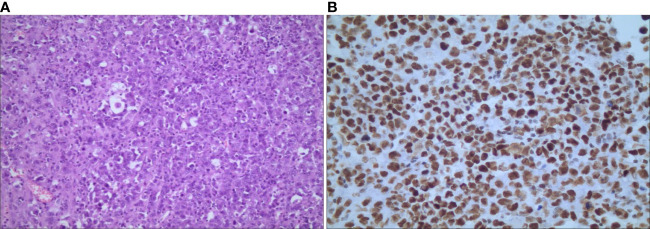
Microscopic findings of primary adrenal epithelioid sarcoma. **(A)** hematoxylin-eosin staining shows that polygonal cells were distributed separately, scattered, in clusters orpatches, with large nuclei and prominent nucleoli (magnification, ×100); **(B)** immunochemical staining shows nuclear positivity for INI-1 (magnification, ×400).

Given the extensive region of focus, laparoscopic right adrenalectomy and intestine adhesion relaxation were performed. Intraoperative findings showed that the right adrenal was occupied by a round-like mass with a diameter of about 10 cm, while the normal adrenal tissue was compressed. Due to the huge size, adhesion of the lesion and liver was obvious and the inferior vena cava was compressed. Pathological examination revealed an incomplete capsule in the adrenal tissue, whose cut surface was gray, reddish-gray and soft. Microscopically, polygonal cells were established to be distributed separately, scattered, in clusters orpatches, with large, vesicular nuclei and prominent nucleoli. Immunohistochemical analysis showed that tumor cells were positive for CK8/18, EMA, vimentin, CD10, Desmin, CD34, SMARCB1/INI-1, SOX-2, SALL4, p53 and Ki-67, while SMARCA4/BRG1, SMARCA 2(BRM), Cr, S-100, HMB-45, Pax-2, Pax-8, WT-1, CK5/6, Melan-A, CK-7, Alpha-inhibin, RCC, MyoD1, Myogenin and SMA were negative (**Figure 3**). Based on the above findings, the patient was diagnosed with right adrenal epithelioid sarcoma. About two months after the operation, PET/CT examination showed recurrence with multiple systemic metastases. Then, the patient underwent chemotherapy of anlotinib combined with anti-PD-1 antibody and epirubicin for 1 cycle. At present, he has a good response, showing the reduction or disappearance of tumor mass, and close follow-up is continuing.

## Discussion and conclusion

Epithelioid sarcoma (ES), a malignant soft tissue sarcoma with a certain degree of epithelization, accounts for < 1% of soft-tissue sarcomas. The two pathological subtypes of ES are classical and proximal types. The conventional type is more common and is often located in superficial dermis or subcutaneous tissues of distal limbs of young adults, whereas the proximal type is quite often located in the proximal soft tissue of elderly patients, such as the limb band, trunk and pelvis. The tumor cells of this subtype are higher grade and demonstrate a rhabaoid phenotype, compared to the classical type.

Because it is highly rare, accurate diagnosis of the primary ES of solid organs by radiologists is difficult. To elucidate on the disease, we report a case of adrenal ES, which is the fifth reported case of ES arising from the adrenal gland.

For our case, the mass was detected during physical examination, without obvious clinical manifestations. In a review of previous CT imaging in 2019, no adrenal mass was found, indicating rapid progress. Reports on MRI and CT of proximal ES involving uterine body, cervix and renal have been relatively few. To date, four cases of primary adrenal epithelioid sarcoma have been reported, but imaging features were not mentioned (3–6). A typical CT of proximal ES reveals slightly lower or equal density mass in homogeneous enhancement on enhanced scan. For the larger, manifestations include tissue invasion, hemorrhage and necrosis, but it is rarely to find calcification (7). On MRI, proximal ES is usually homogeneous in signal, isointense or hyperintense in T_1_WI and T_2_WI, and homogeneous or heterogeneous enhancement, with no enhancement in the necrotic area. There is infiltration to adjacent tissues or bones in some cases (8). In our case, plain CT revealed a slightly low-density mass in the right adrenal gland. Upon MRI, the mass exhibited heterogeneous isointensity in T_1_WI and relative hyperintensity with hypointensity central scar inside in fat-suppression T_2_WI. The mass had hyperintense signals on DWI and hypointense signals on the ADC map, while the central scar presented hypointensity. After Gd-DTPA enhancement scanning, it also showed progressive and slight to moderate enhancement on fat-suppression T_1_WI, with no enhancement of the central scar. The CT findings were comparable to those reported earlier. Due to the small number of reported patients, characteristic imaging findings should be investigated further for comprehensive understanding of the disease. A summary of clinical characteristics and imaging findings of the four reported cases of primary adrenal ES are listed in **Table 1**.

**Table 1:** Case reports of primary ES of adrenal gland.

Author(s)	Age/Gender	Size/Location(R/L)	Symptom	Imaging finding	Diagnosis methods	Laboratory examination	Histology	IHC	Treatment
Alikhan et al. (3),2017	72/M	3.6cm/R	Abdominal pain and nausea	CT and MRI: an incidental adrenal mass	Adrenalectomy	Urine testing for metanephrines, normetanephrines, and cortisol, serum ACTH, renin, and aldosterone were negative.	Cells with abundant eosinophilic cytoplasm and atypical, vesicular nuclei	AE1/AE3+ EMA+ CD34+ CD31+; loss of nuclear INI-1 expression	Laparoscopic adrenalectomy and radiation therapy
Huang et al. (4),2019	31/F	4.4 × 2.9 × 3.3 cm/L	nausea and rectal bleeding.	CT: a heterogenous mass and retroperitoneal lymphadenopathy; MRI: an extensive hypermetabolic mass and left retroperitoneal lymphadenopathy; PET/CT: no additional distant metastasis	CT-guided core biopsy	Functional biochemical workup was negative.	Cells with granular eosinophilic cytoplasm, irregular nuclear borders, and nuclear hyperchromasia with prominent nucleoli.	AE1/AE3+ CK7+ Fli-1+ CD34+; focal CD99+; loss of nuclear expression of INI-1;	Neoadjuvant chemotherapy with ifosfamide and anthracycline
Monappa et al. (5), 2020	11/M	10.8×10.8×13.5cm/R	Right loin pain and abdominal fullness	CT: a well-defined complex cystic lesion with a heterogeneously enhancing solid peripheral component	Tumor excision	Serum lactate dehydrogenase levels were high and 24-hurine vanillylmandelic acid was normal	Cells with hyperchromatic, pleomorphic nuclei, prominent nucleoli and focal alveolar pattern and interstitial hyalinization.	AE1/AE3+vimentin+ CD34+; loss of expression of INI-1	Right suprarenal tumor excision and adrenalectomy
Martinez et al. (6), 2020	82/F	3.3cm/L	right flank pain	CT: an indeterminate mass with an absolute and relative washout of 21.9% and 10.1%	Mass excision	Metabolic workup with a comprehensive metabolic panel, plasma metanephrines, and dexamethasone suppression test was normal.	Cells with moderate eosinophilic cytoplasm and rhabdoid features, associating with prominent lymphoid infiltrate.	Cam 5.2+ CD34+ focal calretinin+; loss of nuclear expression of INI-1;	Adrenalectomy,splenectomy,nephre-ctomyand mass excision.
Present case	69/M	10.6×6.4×11.0cm/R	Incidental finding	CT:a heterogeneous mass; MRI: a heterogeneous mass with hypointensity central scar, and slight to moderate enhancement with no enhancement of central scar	Adrenalectomy	The index of adrenal function laboratory assay was normal	Cells distributed separately, scattered, in clusters or patches, with large, vesicular nuclei and prominent nucleoli.	INI-1+, epithelial markers+, cytokeratins+, vimentin+, and CD34+	Adrenalectomy

Malignant lesions with adrenal masses should be considered in differential ES diagnosis. Adrenocortical adenocarcinoma, the most common primary malignant tumor of the adrenal gland, is usually characterized by irregular, huge and ill-defined mass (>5 mm in diameter), prone to central necrosis, and 20~30% with calcification. Lesion enhancement scan findings revealed a heterogeneous enhancing mass that descended and attenuated slowly (9). Angiosarcoma is a well-defined heterogeneous tumor that is characterized by bleeding and calcification. The necrosis area of angiosarcoma is greater, compared to that of ES. Meanwhile, it has specific imaging features on enhanced CT or MRI: demonstrating peripheral enhancement in arterial phase and expansion to the center in venous and delayed phase, which is similar to typical findings of hemangioma (10, 11). A large mass with intra-tumor necrosis and adjacent structure invasion are common imaging findings of leiomyosarcoma, but bleeding and calcification are rare. The solid portion is persistently enhanced, while the area of cystoid variation and necrosis are not enhanced (12). Visible tumor neovascularization and needlepoint-like blood vessels in the tumor can reflect its characteristics and be of great value for differential diagnosis. ES should also be differentiated from Sarcomatoid carcinoma, which presents as large cystic and solid mass, and evident delayed enhancement of the wall and septa (13).

In previous studies, immunohistochemical analysis of adrenal ES revealed positive expressions of epithelial markers, cytokeratins, vimentin, CD34, and negative expressions of CD31, SMARCB1/INI-1 and S-100. In particular, the loss of SMARCB1/INI-1 is considered to be the most specific aspect of immunohistochemical features for the diagnosis of ES. SMARCB1/INI-1 is one of a component of SW1/SNF chromatin-remodeling complex, which is the main condition factor for regulating gene expression (14). It is noteworthy that SMARCB/INI-1 was positive in our case, while the loss of SMARCB1/INI-1 expression has been detected in all previous reports of adrenal ES. The positive expression of SMARCB1/INI-1 often suggests malignant rhabdoid features, which may be correlated with the core subunit protein deficiency or other abnormalities causing dysfunction of SWI/SNF chromatin remodeling (15). Kohashi et al. (16) found that SMARCB1/INI-1 preserved ES predicts more aggressive biology behaviors than epithelioid sarcoma with loss of SMARCB1/INI-1 expression. Therefore, the expression of SMARCB1/INI-1 may be an important index of poor prognosis of proximal ES.

In conclusion, we report a case of proximal adrenal ES, which is rare and not commonly suspected in clinical practice. However, due to the high aggressiveness, poor prognosis, and prone to recurrence and metastasis, radiotherapy and chemotherapy after radical surgical resection is the optimal treatment. Currently, definite diagnosis of adrenal ES depends on histopathologic and immunohistochemical characteristics, but imaging examination displays some features and plays a significant role in diagnosis and differential diagnosis, providing the relationship with nearby structures. Therefore, awareness of the above may be useful for clinical diagnosis and treatment.

## Data availability statement

The original contributions presented in the study are included in the article/supplementary material. Further inquiries can be directed to the corresponding author.

## Ethics statement

Written informed consent was obtained from the individual(s) for the publication of any potentially identifiable images or data included in this article.

## Author contributions

HY reviewed the literatures and wrote the initial draft of this manuscript. YZ prepared the images and for collected the data of the patient. LW revised the manuscript for English grammar. All authors contributed to the article and approved the submitted version. JZ supervised and reviewed the manuscript.
